# Supplementary material for: Demographic history and biologically relevant genetic variation of Native Mexicans inferred from whole-genome sequencing
Source: Nat Commun. 2017 Oct 18;8:1005. doi: 10.1038/s41467-017-01194-z (PMC5647344; doi:10.1038/s41467-017-01194-z)
Supplement: Supplementary file 1 — Supplementary Information [file 41467_2017_1194_MOESM1_ESM.pdf]

Supplementary Information

Supplementary Table 1. Mean sequence coverage per genome

Sample ID	Fully called genome fraction	>= 5x	10x	20x	30x	40x
Tar1	0.969	0.993	0.983	0.926	0.807	0.632
Tar2	0.966	0.993	0.984	0.939	0.831	0.653
Tep1	0.969	0.993	0.983	0.933	0.825	0.665
Tep2	0.970	0.993	0.983	0.930	0.819	0.657
Nah1	0.970	0.994	0.984	0.934	0.825	0.661
Nah2	0.968	0.994	0.985	0.942	0.842	0.674
Tot1	0.970	0.994	0.984	0.932	0.822	0.658
Tot2	0.968	0.994	0.985	0.943	0.842	0.674
Zap1	0.968	0.994	0.985	0.940	0.838	0.673
Zap2	0.968	0.994	0.985	0.943	0.844	0.681
May1	0.967	0.994	0.985	0.941	0.842	0.680
May2	0.968	0.994	0.986	0.947	0.857	0.703
Mes1	0.967	0.994	0.985	0.939	0.834	0.665
Mes2	0.970	0.994	0.984	0.934	0.826	0.665
Mes3	0.970	0.993	0.983	0.930	0.819	0.657

Tar1 and Tar2 (Tarahumara), Tep1 and Tep2 (Tepehuano), Nah1 and Nah2 (Nahua), Tot1 and Tot2 (Totonaca), Zap1 and Zap2 (Zapoteca), May1 and May2 (Maya) Mes1, Mes2 and Mes3 (Mestizo).

Supplementary Table 2. Total number of SNVs per genome

	SNVs		Coding		Missense		Nonsense		Promoter	
**Sample ID**	**Total**	**Novel (%)**	**Total**	**Novel (%)**	**Total**	**Novel (%)**	**Total**	**Novel (%)**	**Total**	**Novel (%)**
Tar1	3215310	57731 (1.8)	20122	429 (2.1)	8947	291 (3.3)	69	4 (5.8)	130443	2584 (2.0)
Tar2	3220342	61245 (1.9)	20222	474 (2.3)	8921	293 (3.3)	80	7 (8.8)	130172	2829 (2.2)
Tep1	3248361	56141 (1.7)	20199	408 (2.0)	9090	261 (2.9)	71	2 (2.8)	130833	2508 (1.9)
Tep2	3248073	56663 (1.7)	20160	421 (2.1)	8927	270 (3.0)	76	6 (7.9)	131631	2516 (1.9)
Nah1	3224763	57646 (1.8)	20261	475 (2.3)	8995	316 (3.5)	82	8 (9.8)	130971	2510 (1.9)
Nah2	3237628	57951 (1.8)	20231	484 (2.4)	8967	307 (3.4)	80	8 (10.0)	132536	2483 (1.9)
Tot1	3230580	56526 (1.7)	20061	438 (2.2)	8927	278 (3.1)	82	6 (7.3)	131038	2627 (2.0)
Tot2	3243532	57543 (1.8)	20081	493 (2.5)	8942	320 (3.6)	88	6 (6.8)	130601	2533 (1.9)
Zap1	3228709	56809 (1.8)	20138	454 (2.3)	9076	302 (3.3)	88	7 (8.0)	131483	2544 (1.9)
Zap2	3244724	56504 (1.7)	20382	471 (2.3)	9086	294 (3.2)	80	6 (7.5)	130221	2564 (2.0)
May1	3260290	57596 (1.8)	20471	448 (2.2)	9086	290 (3.2)	85	10 (11.8)	131981	2418 (1.8)
May2	3260903	59011 (1.8)	20316	486 (2.4)	9027	298 (3.3)	76	10 (13.2)	132161	2580 (2.0)
Mes1	3424588	57380 (1.7)	21400	434 (2.0)	9600	276 (2.9)	81	9 (11.1)	138700	2541 (1.8)
Mes2	3450123	58817 (1.7)	21713	398 (1.8)	9787	233 (2.4)	84	7 (8.3)	141410	2702 (1.9)
Mes3	3426187	57268 (1.7)	21847	425 (1.9)	9765	258 (2.6)	79	5 (6.3)	130443	2584 (2.0)

The novel, coding, missense and nonsense SNVs were identified from the master variation files. Tar1 and Tar2 (Tarahumara), Tep1 and Tep2 (Tepehuano), Nah1 and Nah2 (Nahua), Tot1 and Tot2 (Totonaca), Zap1 and Zap2 (Zapoteca), May1 and May2 (Maya) Mes1, Mes2 and Mes3 (Mestizo).

Supplementary Table 3. Proportion of missense SNVs predicted to be damaging by PolyPhen-2

Sample ID*	Missense SNVs	Missense SNVs predicted as damaging	Novel Missense SNVs	Novel Missense SNVs predicted as damaging
Tar1	8947	1293 (14.5)	291	77 (26.5)
Tar2	8921	1370 (15.4)	293	100 (34.1)
Tep1	9090	1324 (14.6)	261	74 (28.4)
Tep2	8927	1323 (14.8)	270	84 (31.1)
Nah1	8995	1318 (14.7)	316	83 (26.3)
Nah2	8967	1331 (14.8)	307	95 (30.9)
Tot1	8927	1236 (13.8)	278	76 (27.3)
Tot2	8942	1345 (15.0)	320	100 (31.3)
Zap1	9076	1322 (14.6)	302	92 (30.5)
Zap2	9086	1316 (14.5)	294	80 (27.2)
May1	9086	1314 (14.5)	290	77 (26.6)
May2	9027	1339 (14.8)	298	101 (33.9)
Mes1	9600	1442 (15.0)	276	92 (33.3)
Mes2	9787	1495 (15.3)	233	74 (31.8)

*Only unrelated Individuals were included in the analysis. Mes3 was excluded for being related to Mes1 and Mes2. Tar1 and Tar2 (Tarahumara), Tep1 and Tep2 (Tepehuano), Nah1 and Nah2 (Nahua), Tot1 and Tot2 (Totonaca), Zap1 and Zap2 (Zapoteca), May1 and May2 (Maya) Mes1 and Mes2 (Mestizo).

Supplementary Table 4. Proportion of Insertions (INS) and Deletions (DEL) per genome

Sample ID	INS	Novel INS (%)	DEL	Novel DEL (%)
Tar1	211905	28590 (13.5)	215129	21590 (10.0)
Tar2	206832	29275 (14.2)	212070	24673 (11.6)
Tep1	208685	27565 (13.2)	212117	20849 (9.8)
Tep2	212401	28664 (13.5)	214888	21344 (9.9)
Nah1	214900	29235 (13.6)	217933	22408 (10.3)
Nah2	213346	29234 (13.7)	215657	21539 (10.0)
Tot1	214246	29084 (13.6)	217153	21685 (10.0)
Tot2	213038	29482 (13.8)	214717	21500 (10.0)
Zap1	212397	29354 (13.8)	215062	21928 (10.2)
Zap2	213040	29220 (13.7)	215609	21966 (10.2)
May1	213601	29403 (13.8)	216960	22469 (10.4)
May2	214714	29185 (13.6)	218852	23548 (10.8)
Mes1	220897	30534 (13.8)	223440	23621 (10.6)
Mes2	224015	31064 (13.9)	228618	24643 (10.8)
Mes3	224354	31377 (14.0)	227078	24324 (10.7)

Tar1 and Tar2 (Tarahumara), Tep1 and Tep2 (Tepehuano), Nah1 and Nah2 (Nahua), Tot1 and Tot2 (Totonaca), Zap1 and Zap2 (Zapoteca), May1 and May2 (Maya) Mes1, Mes2 and Mes3 (Mestizo).

Supplementary Table 5. Copy number Variations (CNVs) and mobile element insertions (MEIs) per genome

Sample ID	Total bases comprising CNVs	CNV count	Novel CNVs (%)	MEI count	Novel MEIs (%)
Tar1	5051458	257	12 (4.7)	3904	2990 (76.6)
Tar2	4766831	237	11 (4.6)	4005	3060 (76.4)
Tep1	4738934	232	11 (4.7)	3794	2876 (75.8)
Tep2	4647674	238	10 (4.2)	3594	2652 (73.8)
Nah1	5781169	262	21 (8.0)	4073	3104 (76.2)
Nah2	5047028	241	11 (4.6)	3768	2830 (75.1)
Tot1	4902104	247	12 (4.9)	3879	2956 (76.2)
Tot2	5112833	248	11 (4.4)	3783	2811 (74.3)
Zap1	4685479	225	9 (4.0)	3706	2750 (74.2)
Zap2	4873479	237	14 (5.9)	3730	2783 (74.6)
May1	6109479	250	17 (6.8)	3782	2848 (75.3)
May2	5609896	256	11 (4.3)	3683	2736 (74.3)
Mes1	6081028	261	10 (3.8)	3845	2822 (73.4)
Mes2	5746104	254	8 (3.1)	3896	2860 (73.4)
Mes3	5972104	270	7 (2.6)	3936	2909 (73.9)

Tar1 and Tar2 (Tarahumara), Tep1 and Tep2 (Tepehuano), Nah1 and Nah2 (Nahua), Tot1 and Tot2 (Totonaca), Zap1 and Zap2 (Zapoteca), May1 and May2 (Maya) Mes1, Mes2 and Mes3 (Mestizo).

Supplementary Table 6. Homozygous and heterozygous SNV fraction per genome

Sample ID	SNVs	Homozygous SNVs (%)	Heterozygous SNVs (%)
Tar1	3215310	1458278 (45.4)	1625670 (50.6)
Tar2	3220342	1509974 (46.9)	1646333 (51.1)
Tep1	3248361	1425111 (43.9)	1694530 (52.2)
Tep2	3248073	1435195 (44.2)	1683660 (51.8)
Nah1	3224763	1458995 (45.2)	1634261 (50.7)
Nah2	3237628	1496420 (46.2)	1680857 (51.9)
Tot1	3230580	1431641 (44.3)	1668339 (51.6)
Tot2	3243532	1485662 (45.8)	1697952 (52.3)
Zap1	3228709	1492634 (46.2)	1675910 (51.9)
Zap2	3244724	1497361 (46.1)	1687953 (52.0)
May1	3260290	1483623 (45.5)	1716113 (52.6)
May2	3260903	1497706 (45.9)	1704023 (52.3)
Mes1	3424588	1319114 (38.5)	2046515 (59.8)
Mes2	3450123	1243415 (36.0)	2081594 (60.3)
Mes3	3426187	1257320 (36.7)	2039962 (59.5)

Tar1 and Tar2 (Tarahumara), Tep1 and Tep2 (Tepehuano), Nah1 and Nah2 (Nahua), Tot1 and Tot2 (Totonaca), Zap1 and Zap2 (Zapoteca), May1 and May2 (Maya) Mes1, Mes2 and Mes3 (Mestizo).

Supplementary Table 7. Potentially Pathogenic Alleles identified in the 12 NA genomes

Chr	Position	rsID	Alt/Ref	CEU	CHB & JPT	12G	312 NA	Trait	EFO category
1	158971086	rs4657616	G/A	0.4	0.45	0.92	---	Hematology traits	Hematological measurement
2	46205039	rs13396424	G/T	0.17	0.58	0.96	---	Metabolite levels (X-11787)	Other measurement
2	191902758	rs3821236	A/G	0.19	0.41	0.96	0.77	Systemic sclerosis	Immune system disease
2	102851708	rs2302612	C/T	0.18	0.27	0.92	---	Serum protein levels (sST2)	Other measurement
2	42276921	rs4952590	T/C	0.1	0.45	0.88	---	Atopy	Other disease
2	74939176	rs1137	C/T	0.81	0.19	0.83	0.70	Myopia	Other disease
2	46921285	rs12474201	A/G	0.32	0.12	0.79	---	Height	Body measurement
3	121793187	rs4308217	A/C	0.34	0.11	0.92	0.81	Multiple sclerosis	Immune system disease
3	71055162	rs13093086	A/G	0.51	0.02	0.75	---	Intraocular pressure	Other measurement
4	100239319	rs1229984	C/T	0.98	0.27	1	---	Upper aerodigestive tract cancers	Cancer
4	79280693	rs1268789	C/T	0.67	0.28	0.67	---	Hair morphology	Other trait
5	155302582	rs1432723	A/G	0.22	0.18	1	---	Obesity-related traits	Other measurement
6	43925526	rs4513773	G/A	0.47	0.29	0.92	0.76	Vascular endothelial growth factor (VEGF) levels	Other measurement
6	50845490	rs2207139	G/A	0.18	0.17	0.67	---	Obesity	Metabolic disease
7	133335176	rs10488172	G/T	0.23	0.48	1	---	Tonometry	Other measurement
7	32347335	rs215614	A/G	0.66	0.35	0.92	---	Smoking behavior	Biological process
8	60961821	rs569688	T/G	0.23	0.34	0.96	0.85	Myopia	Other disease
9	113300835	rs1889321	T/C	0.69	0.27	0.92	---	Pulmonary function decline	Other measurement
10	34231275	rs7905537	C/A	0.29	0.24	0.88	0.76	Emphysema-related traits	Other disease
11	103660567	rs974819	C/T	0.76	0.38	0.96	---	Coronary heart disease	Cardiovascular disease
11	61597212	rs174570	T/C	0.16	0.41	0.92	0.92	LDL cholesterol	Other measurement
11	131336074	rs992564	T/G	0.05	0.53	0.79	0.65	Bipolar disorder and schizophrenia	Other disease
11	123361397	rs735665	A/G	0.17	0.01	0.79	---	Chronic lymphocytic leukemia	Cancer
12	110398145	rs4766646	A/T	0.68	0.31	1	---	Metabolite levels (MHPG)	Other measurement
12	4416304	rs4625554	G/A	0.3	0.3	0.96	---	Major depressive disorder	Other disease
13	21209512	rs7326068	A/G	0.2	0.7	1	0.84	Schizophrenia, bipolar disorder and depression	Other disease
13	50986118	rs201789	C/T	0.74	0.21	0.83	---	Anthropometric traits	Body measurement

15	45934869	rs607541	A/C	0.15	0.64	0.96	---	Obesity-related traits	Other measurement
15	78908032	rs8042374	G/A	0.23	0.73	0.96	---	Lung cancer	Cancer
15	78863472	rs667282	C/T	0.24	0.48	0.96	---	Smoking behavior	Biological process
15	48392165	rs1834640	G/A	0.01	0.92	0.92	---	Skin pigmentation	Other trait
15	68043057	rs8028313	G/C	0.23	0.5	0.92	---	Obesity	Metabolic disease
15	68086838	rs2241423	A/G	0.23	0.6	0.92	---	Body mass index	Body measurement
15	57910164	rs937254	A/G	0.4	0.52	0.83	0.84	Coronary heart disease	Cardiovascular disease
16	11347858	rs4780355	C/T	0.31	0.75	1	---	Crohn's disease and psoriasis	Digestive system disease
16	792190	rs11648796	G/A	0.29	0.79	0.96	---	Height	Body measurement
16	11364079	rs416603	T/A	0.44	0.13	0.92	0.75	Type 1 diabetes	Immune system disease
19	3797100	rs12104221	T/C	0.11	0.17	0.88	---	Obesity-related traits	Other measurement
20	62318220	rs4809324	C/T	0.1	0.13	0.79	---	Glioma (high-grade)	Cancer
22	42218856	rs7364180	G/A	0.23	0.28	1	---	Alzheimer's disease biomarkers	Nervous system disease
22	41431342	rs4820425	A/C	0.31	0.06	1	---	Crohn's disease	Digestive system disease
22	41661154	rs2235852	T/G	0.35	0.5	1	0.92	Self-reported allergy	Immune system disease
22	21964761	rs4821112	A/G	0.2	0.48	0.79	---	Mean corpuscular volume	Hematological measurement
X	133322604	rs765132	T/C	0.05	0	0.88	0.59	Response to anti-TNF alpha therapy in inflammatory bowel disease	Response to drug

CEU (Utah residents with Northern and Western European ancestry), CHB (Han Chinese), JPT (Japanese), 12G (12 Native American genomes); 312 NA (Native Americans with microarray genotyping data); EFO (Experimental factor ontology).

Supplementary Table 8. Native Mexican Populations from Moreno-Estrada *et al*. 2014[1]

ID	Population	N
SER	Seri	21
TAR	Tarahumara	24
TEP	Tepehuano	23
HUI	Huichol	24
PUR	Purépecha	23
NAH	Nahua	69
TOT	Totonaca	23
MAZ	Mazateca	17
TRI	Triqui	24
ZAP	Zapoteca	44
MAY	Maya	45
TZO	Tzotzil	21
TOJ	Tojolabal	21
LAC	Lacandona	22

Supplementary Table 9. Concordance of genome sequencing and microarray genotyping

Sample ID	Number of genotypes called	Concordant genotypes (%)
Tar1	716638	712266 (99.4)
Tar2	716425	706768 (98.7)
Tep1	717389	702015 (97.9)
Tep2	717052	703919 (98.2)
Nah1	716791	711750 (99.3)
Nah2	717278	711844 (99.2)
Tot1	717124	711693 (99.2)
Tot2	716628	712352 (99.4)
Zap1	716530	714787 (99.8)
Zap2	716943	716411 (99.9)
May1	716724	716155 (99.9)
May2	717311	716813 (99.9)
Mes1	727948	722454 (99.2)
Mes2	727948	721769 (99.2)
Mes3	727948	722430 (99.2)

Tar1 and Tar2 (Tarahumara), Tep1 and Tep2 (Tepehuano), Nah1 and Nah2 (Nahua), Tot1 and Tot2 (Totonaca), Zap1 and Zap2 (Zapoteca), May1 and May2 (Maya) Mes1, Mes2 and Mes3 (Mestizo).

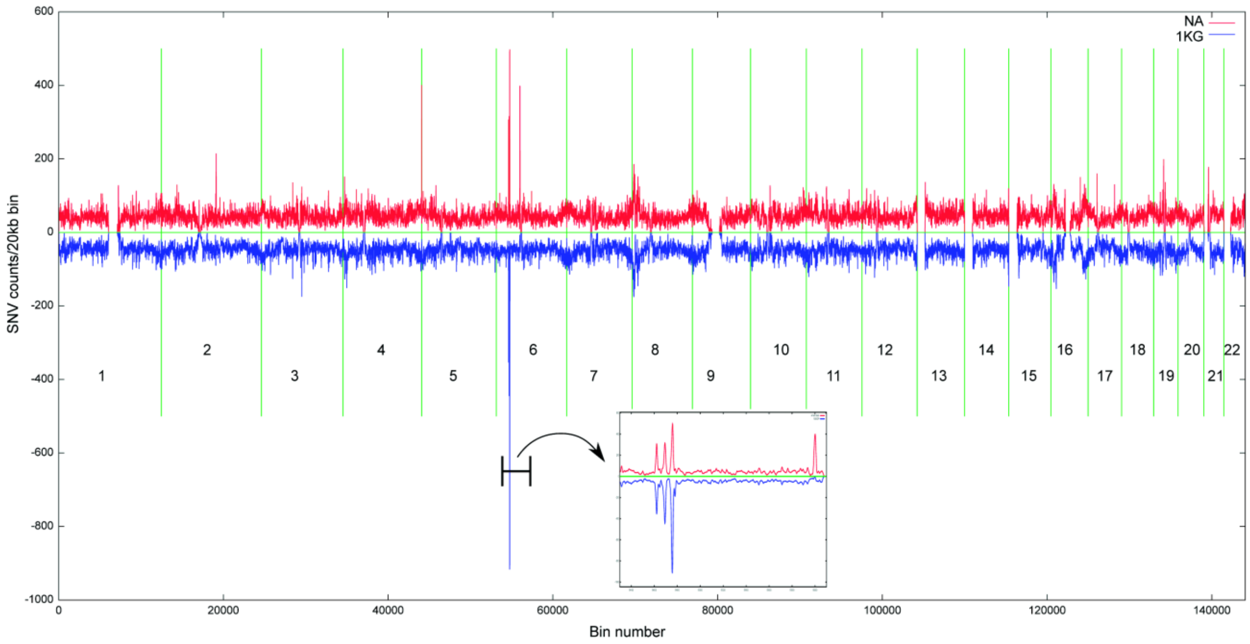

Supplementary Figure 1. Genome-wide variability. Average number of SNVs per individual for across the genome. The red line represents the 12 Native American genomes and the blue line represents genomes from the 1KG project. Chromosome 6 contains three variability peaks shared by both populations, and one peak that is private to NA, indicated with an arrow.

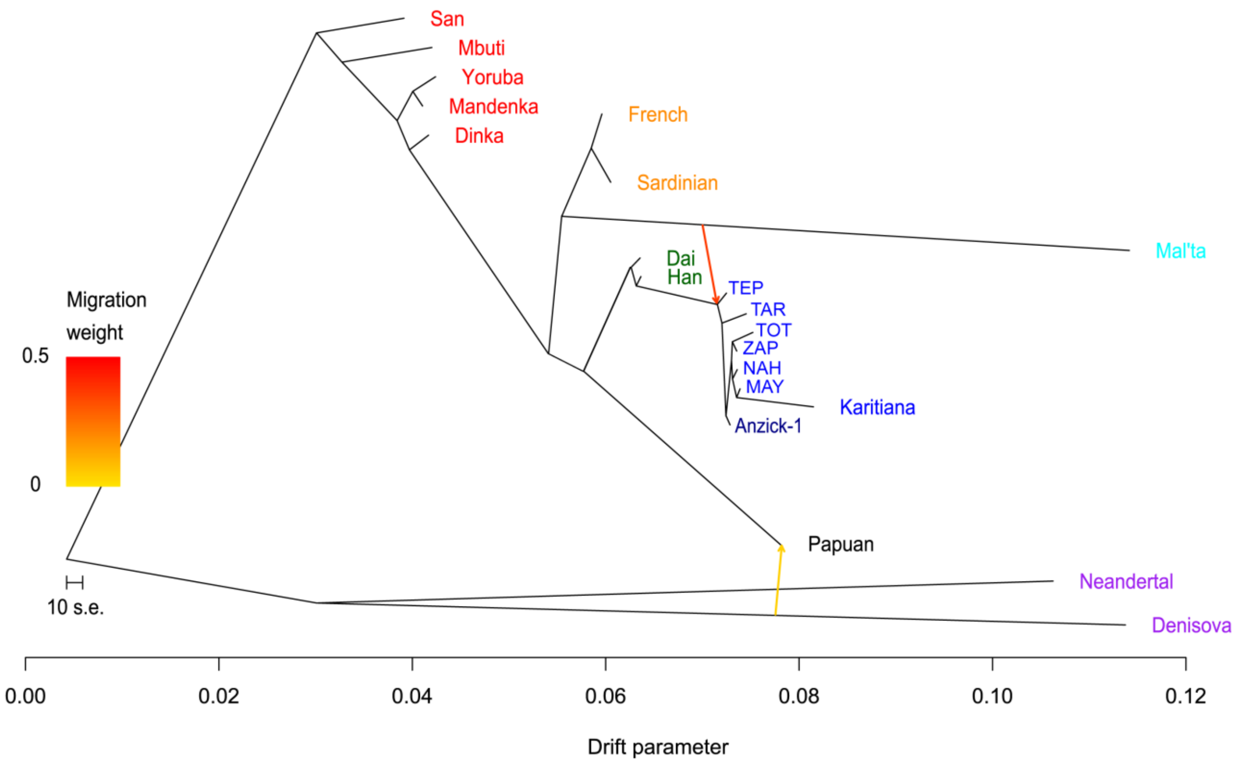

Supplementary Figure 2. Maximum likelihood population tree using sequencing data from 12 NA genomes (TAR, Tarahumara; TEP, Tepehuano; NAH, Nahua; TOT, Totonaca; ZAP, Zapoteca and MAY, Maya), 11 genomes from worldwide populations (San, Mbuti, Yoruba, Mandenka, Dinka, French, Sardinian, Dai, Han, Karitiana and Papuan) and 4 ancient individuals (Neanderthal, Denisovan, Anzick-1, and the Mal'ta child). The plot shows splitting of the 12 NA individuals inferred by TreeMix. Gene flow between these lineages is indicated by arrows. The scale bar shows ten times the average standard error (s.e.) of the entries in the sample covariance matrix.

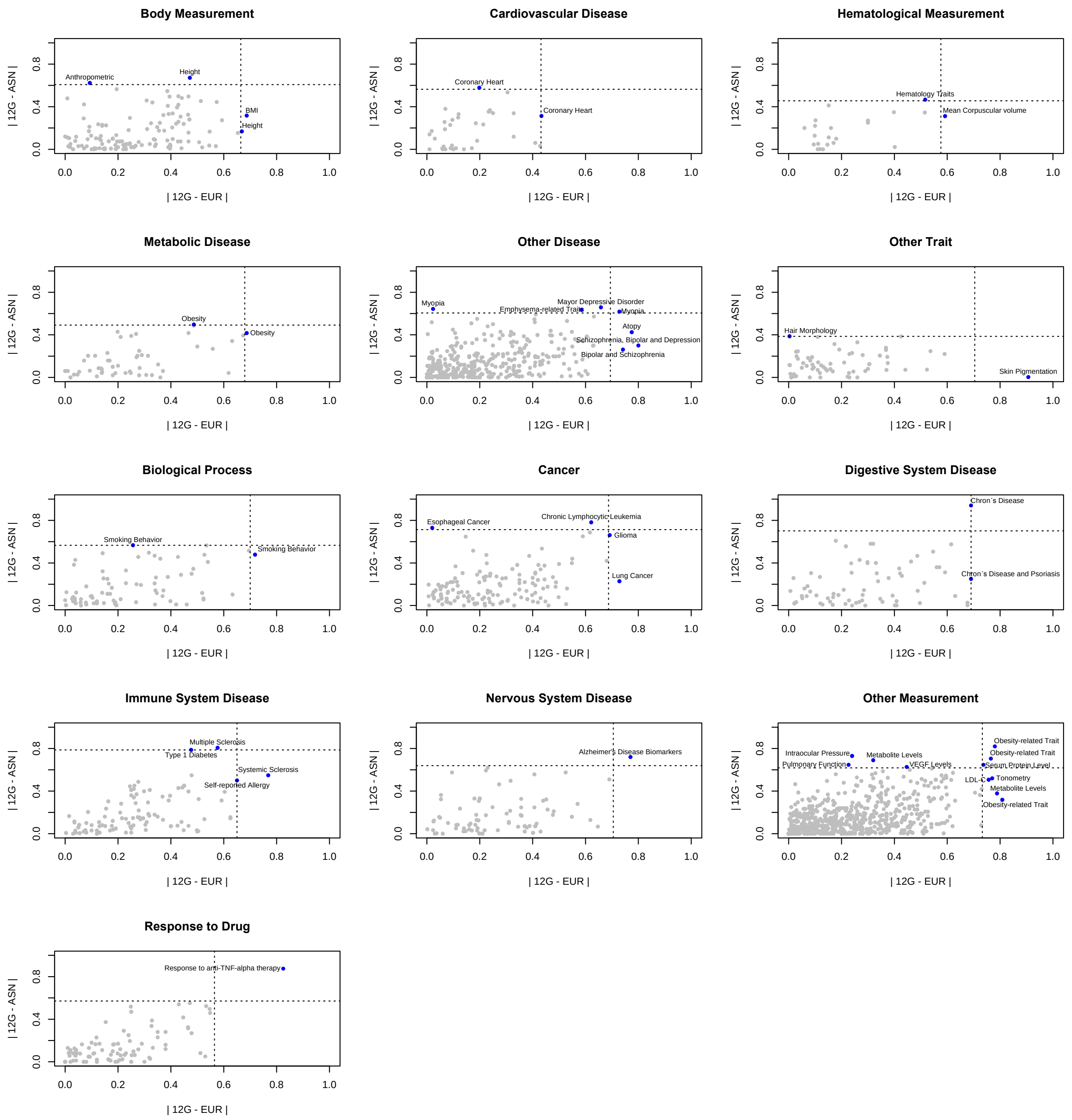

Supplementary Figure 3. Comparison of GWAS variant allele frequencies between NAs and continental populations. Scatterplots including variants associated with biological traits in the GWAS catalog shared by the 12 NA individuals, grouped according to their Experimental Factor Ontology (EFO). Each dot represents the difference of allele frequencies between the 12 genomes and CEU (EUR, x-axis) or CHB and JPT (ASN, y-axis) populations from 1KG project. Blue dots indicate variants exceeding the 99th quantile threshold in each EFO category (horizontal and vertical dash lines).

a. Mayas

	Missense + Promoter							Missense							Promoter						
	MES	TEP	ZAP	MAY	NAH	TOT	TAR	MES	TEP	ZAP	MAY	NAH	TOT	TAR	MES	TEP	ZAP	MAY	NAH	TOT	TAR
**Nervous System Diseases**		6.9E-06	1E-05	4E-08		0.0043	2E-05		0.0005	0.0055	0.002			0.0025			0.0024	1.2E-06		0.0023	0.0043
**Neurologic Manifestatio**		0.0005	0.0072	3E-06		0.0004	0.009						0.0072				0.0048	4.9E-06			
**Disease Susceptibility**	1.6E-06	1.2E-07	0.0004	4.9E-06	1.4E-10	0.0002	0.001	0.001	0.0006		7E-07	6.8E-08	0.0058	0.0065	0.0028	0.0007	0.0012		0.0065		
**Mitochondrial Diseases**				7.4E-06														4.9E-08			
**Mental Disorders**		0.0003	0.0024	8.5E-06		0.0026			0.0025		8E-04									0.0049	
**Adhesion**	5.2E-06	6.1E-08	0.0024	1.1E-05	3.4E-07	3E-10	5E-10	8E-05	2E-05	0.001	2E-05	3E-06	4E-08	3.5E-07						0.0091	0.0019
**Congenital Abnormalities**	0.0016	3.5E-08	1E-06	2.7E-05	0.0009	2E-09	1E-06		2E-05	0.0005	0.001	0.0014	3E-07	0.0004	0.0059	0.0068	0.0055			0.0089	
**Genetic Predisposition to Disease**	2.2E-07	6.6E-06	1E-04	2.9E-05	3.4E-11	7E-05	4E-05	3E-04	0.005		1E-05	1.9E-08	0.0016	0.0008	0.0013	0.0016	0.0001		0.0065		
**Neurodegenerative Diseases**	0.0094			2.9E-05														0.0004			
**Pancreatic Diseases**	0.0024		0.0072	3.5E-05			0.005	0.01			8E-04										
**Vascular Diseases**	5.6E-07	0.0064	1E-05	5E-05	0.00002		4E-04	4E-04		0.0017		0.0014			0.0028		0.0024	0.0002	0.0065		
**Urogenital Neoplasms**		0.0005	1E-04	6.4E-05					0.0077	0.0055							0.0061	0.0006			
**Chromosome Aberratio**				7.7E-05		0.0057	0.002				0.002			0.005		0.0093					
**Dementia**				8E-05		0.0091												0.0005			
**Schizophrenia**		0.0015		0.0002		0.0083					0.001										
**Brain Diseases**		0.0018		0.0003											0.0028			0.0044			
**Growth Disorders**				0.0003		0.0015	0.002				0.005									0.0016	0.0061
**Cancer or viral infectio**	0.0003	6.1E-08	9E-05	0.0004	1.7E-05	1E-05	6E-06		0.0003			0.0059	0.002	0.0002	0.0038	0.0003	0.0012	0.0035	0.0014	0.0023	
**Neoplasms**	0.0063	8.5E-07	0.0014	0.0004	2.2E-05	7E-05	9E-05		0.005				0.0024	0.0007		0.0001	0.0024	0.0044	0.0009		
**Musculoskeletal Diseases**		0.001		0.0004	0.0023	1E-05	4E-09						0.0034	0.0004				0.0022	0.0065	0.0009	1E-05
**Musculoskeletal Abnormalities**		0.0035		0.0004		0.0002	0.006				0.003		0.0001								
**Epithelial cancers**	0.0016	0.0064		0.0004		0.0015	0.004	0.007													
**Pancreatic Neoplasms**				0.0004		0.0026	0.001							0.005						0.0087	
**Heart Diseases**	0.0024	0.0073	0.0054	0.0005	2.6E-05	0.0002	9E-05				0.003		0.0034	0.0097					0.0011		0.0035
**Cerebral Infarction**	0.0009			0.0005	0.0038		0.009	0.009				0.0059									
**Stroke**	0.0009			0.0005	0.0013		0.009	0.009				0.0014									
**Stroke NOS**	0.0009			0.0005	0.0013		0.009	0.009				0.0014									
**Carcinoma, Pancreatic Ductal**	0.0049			0.0005							0.005										
**Muscle Weakness**				0.0005		0.0014	0.002										0.0024	5.7E-05	0.0022	0.0062	
**Psychomotor epilepsy**				0.0006							0.003				0.0074						

b. Totonacas

	Missense + Promoter							Missense							Promoter						
	MES	TEP	ZAP	MAY	NAH	TOT	TAR	MES	TEP	ZAP	MAY	NAH	TOT	TAR	MES	TEP	ZAP	MAY	NAH	TOT	TAR
**Adhesion**	5.2E-06	6.1E-08	0.0024	1.06E-05	3.4E-07	2.9E-10	5.36E-10	7.8E-05	1.6E-05	0.001	1.8E-05	3E-06	3.5E-08	3.5E-07						0.0091	0.0019
**Congenital Abnormalities**	0.0016	3.5E-08	1.4E-06	2.66E-05	0.0009	2.3E-09	1.46E-06		1.6E-05	5E-04	0.0013	0.001	3.2E-07	0.0004	0.0059	0.0068	0.0055			0.0089	
**Syndrome**	0.0059	0.0015	0.0084	0.0013	0.0002	1.1E-08	9.49E-05				0.0031	4E-04	5.4E-06	0.0008						0.0034	
**Musculoskeletal Diseases**		0.001		0.0004	0.0023	1.1E-05	4.49E-09						0.0034	0.0004				0.0022	0.0065	0.0009	1.3E-05
**Cancer or viral infectio**	0.0003	6.1E-08	8.8E-05	0.0004	1.7E-05	1.2E-05	5.74E-06		0.0003			0.006	0.002	0.0002	0.0038	0.0003	0.0012	0.0035	0.0014	0.0023	
**Neoplastic Processes**	0.0004	0.0003	0.0096			1.4E-05	0.0008	0.0031	0.0028				0.0034	0.0004						0.0015	
**Chromosome Disorders**		0.0047		0.0025		1.7E-05	0.0017						0.0082							0.0007	
**H Syndrome**		0.002	0.0031		0.0058	2.1E-05	0.000026					0.004	0.0002	0.0007							
**Death, Sudden**	0.0043				0.0076	3.3E-05	0.003						0.001	0.0097							
**Leukemia, Experimental**						3.3E-05														0.0009	
**Death, Sudden, Cardiac**	0.0075					4.7E-05	0.0036						0.0016								
**Genetic Predisposition to Disease**	2.2E-07	6.6E-06	9.6E-05	2.93E-05	3.4E-11	6.7E-05	4.25E-05	0.0003	0.005		1.2E-05	2E-08	0.0016	0.0008	0.0013	0.0016	0.0001		0.0065		
**Neoplasms**	0.0063	8.5E-07	0.0014	0.0004	2.2E-05	7.2E-05	9.49E-05		0.005				0.0024	0.0007		0.0001	0.0024	0.0044	0.0009		
**Tachycardia**	0.0016					9.4E-05		0.0055					0.0014								
**Drug interaction with drug**	0.0016					0.0001	0.0086								0.0028					0.0016	
**Atrioventricular block NOS**						0.0001							7.2E-05								
**Disease Susceptibility**	1.6E-06	1.2E-07	0.0004	4.9E-06	1.4E-10	0.0002	0.001	0.001	0.0006		7.3E-07	7E-08	0.0058	0.0065	0.0028	0.0007	0.0012		0.0065		
**Heart Diseases**	0.0024	0.0073	0.0054	0.0005	2.6E-05	0.0002	9.49E-05				0.0031		0.0034	0.0097					0.0011		0.0035
**Musculoskeletal Abnormalities**		0.0035		0.0004		0.0002	0.0057				0.0031		0.0001								
**Neoplasm Invasiveness**	0.0008		0.0099			0.0002							0.0065							0.0091	
**Ascites**						0.0002							0.0024								
**Leukemia**					0.0013	0.0003							0.0034						0.0065		
**Deglutition Disorders**	0.0016	0.002	0.0007	0.0091	0.0023	0.0003	0.0004	0.0066	0.0066	5E-04	0.0094	8E-04	0.0014	0.0014							
**Leukemia, Myeloid**		0.0073			0.0023	0.0003							0.0003								
**Syncope**	0.0049	0.0016				0.0003							0.0034								
**Nervous System Malformatio**	0.0007					0.0003							0.0016		0.0059		0.0048				
**Acquired Immunodeficiency Syndrome**						0.0003														0.0009	
**Heart Block**						0.0003							0.001								
**Gastroesophageal Reflux**	0.0043	0.0017	0.0031	0.0064	0.0074	0.0004	0.0017		0.0028	0.009	0.0031	0.003	0.001	0.0025							
**Neurologic Manifestatio**		0.0005	0.0072	2.97E-06		0.0004	0.0086						0.0072				0.0048	5E-06			

c. Nahuas

	Missense + Promoter							Missense							Promoter						
	MES	TEP	ZAP	MAY	NAH	TOT	TAR	MES	TEP	ZAP	MAY	NAH	TOT	TAR	MES	TEP	ZAP	MAY	NAH	TOT	TAR
**Genetic Predisposition to Disease**	2.2E-07	6.6E-06	9.6E-05	2.9E-05	3.4E-11	6.7E-05	4.3E-05	0.0003	0.005		1.2E-05	1.9E-08	0.0016	0.0008	0.0013	0.0016	0.0001		0.0065		
**Disease Susceptibility**	1.6E-06	1.2E-07	0.0004	4.9E-06	1.4E-10	0.0002	0.001	0.001	6E-04		7.3E-07	6.8E-08	0.0058	0.0065	0.0028	0.0007	0.0012		0.0065		
**Male Urogenital Diseases**	0.0016		0.0064	0.0064	7.7E-08			0.0066				1.4E-06									
**Urologic Diseases**	0.0024				7.7E-08			0.0035				9.6E-08									
**Adhesion**	5.2E-06	6.1E-08	0.0024	1.1E-05	3.4E-07	2.9E-10	5.4E-10	8E-05	2E-05	0.001	1.8E-05	3E-06	4E-08	4E-07						0.0091	0.0019
**Cardiovascular Diseases**	5.2E-06	0.001	0.00009	0.0008	1.1E-06	0.0015	0.0004	0.0035	0.008	0.0025		0.0011			0.0028			0.0049	0.0009	0.0034	0.0043
**Autoimmune Diseases**	0.0024	0.0083			2.4E-06							1.7E-05			0.0028						
**Kidney Diseases**	0.0009			0.0079	2.6E-06			0.0019				1.7E-05									
**Kidney Failure**					2.6E-06							8.1E-07									
**Immune System Diseases**	0.0024	0.002			6.7E-06	0.006	0.0076					0.0014							0.0022		
**Respiratory Tract Infectio**	0.0049			0.0086	8.1E-06						0.0031	1.8E-05									
**Common Cold**	0.0024			0.0089	8.4E-06			0.0055			0.0031	1.8E-05									
**Glomerular disease**					1E-05							8.1E-07									
**Bacterial Infectio**					1.1E-05	0.0036						0.001					0.0091		0.007	0.0062	
**Glomerulonephritis**	0.0091				1.2E-05							1.4E-06									
**Cancer or viral infectio**	0.0003	6.1E-08	8.8E-05	0.0004	1.7E-05	1.2E-05	5.7E-06		3E-04			0.0059	0.002	0.0002	0.0038	0.0003	0.0012	0.0035	0.0014	0.0023	
**Bronchitis**	0.0043			0.0079	1.8E-05		0.0091				0.0069	1.7E-05									
**Vascular Diseases**	5.6E-07	0.0064	1.1E-05	5E-05	0.00002		0.0004	0.0004		0.0017		0.0014			0.0028		0.0024	0.0002	0.0065		
**Myocardial Infarction**	0.0005				2.1E-05			0.0023				0.0002									
**Neoplasms**	0.0063	8.5E-07	0.0014	0.0004	2.2E-05	7.2E-05	9.5E-05		0.005				0.0024	0.0007		0.0001	0.0024	0.0044	0.0009		
**Heart Diseases**	0.0024	0.0073	0.0054	0.0005	2.6E-05	0.0002	9.5E-05				0.0031		0.0034	0.0097					0.0011		0.0035
**Chronic Disease**					2.7E-05							5.2E-05									
**Preterm rupture of membranes**					8E-05		0.0072					0.0001									
**Ciliary Motility Disorders**					8.6E-05							0.0001									
**Chorioamnionitis**					0.0001		0.0036					0.0002									
**Bronchial Diseases**					0.0001							1.7E-05									
**Fatty Liver**					0.0001														0.0052		
**Syndrome**	0.0059	0.0015	0.0084	0.0013	0.0002	1.1E-08	9.5E-05				0.0031	0.0004	5E-06	0.0008						0.0034	
**Leukemia, Myeloid, Acute**				0.0089	0.0002	0.0015						0.0084	0.0024								
**Endocrine gland disease NOS**		0.002			0.0002							0.001				0.0023					

d. Zapotecas

	Missense + Promoter							Missense							Promoter						
	MES	TEP	ZAP	MAY	NAH	TOT	TAR	MES	TEP	ZAP	MAY	NAH	TOT	TAR	MES	TEP	ZAP	MAY	NAH	TOT	TAR
**Congenital Abnormalities**	0.0016	3.5E-08	1.4E-06	2.7E-05	0.0009	2.3E-09	1.5E-06		1.6E-05	0.0005	0.0013	0.0014	3.2E-07	0.0004	0.0059	0.0068	0.0055			0.0089	
**Nervous System Diseases**		6.9E-06	1.1E-05	4E-08		0.0043	2E-05		0.0005	0.0055	0.0021			0.0025			0.0024	1E-06		0.0023	0.0043
**Vascular Diseases**	5.6E-07	0.0064	1.1E-05	5E-05	0.00002		0.0004	0.0004		0.0017		0.0014			0.0028		0.0024	0.0002	0.0065		
**Protein Deficiency**		0.0035	3.4E-05						0.0026	0.0087							0.0024				
**Eye Diseases**			4.7E-05	0.0049	0.0032	0.0096				0.0021		0.0059									
**cancer or viral infectio**	0.0003	6.1E-08	8.8E-05	0.0004	1.7E-05	1.2E-05	5.7E-06		0.0003			0.0059	0.002	0.0002	0.0038	0.0003	0.0012	0.0035	0.0014	0.0023	
**Cardiovascular Diseases**	5.2E-06	0.001	0.00009	0.0008	1.1E-06	0.0015	0.0004	0.0035	0.0077	0.0025		0.0011			0.0028			0.0049	0.0009	0.0034	0.0043
**Genetic Predisposition to Disease**	2.2E-07	6.6E-06	9.6E-05	2.9E-05	3.4E-11	6.7E-05	4.3E-05	0.0003	0.005		1.2E-05	1.9E-08	0.0016	0.0008	0.0013	0.0016	0.0001		0.0065		
**Urogenital Neoplasms**		0.0005	9.6E-05	6.4E-05					0.0077	0.0055							0.0061	0.0006			
**Disease Susceptibility**	1.6E-06	1.2E-07	0.0004	4.9E-06	1.4E-10	0.0002	0.001	0.001	0.0006		7.3E-07	6.8E-08	0.0058	0.0065	0.0028	0.0007	0.0012		0.0065		
**Lung Neoplasms**		0.0004	0.0004				9.5E-05							0.0018		0.0059	0.0024				
**Deglutition Disorders**	0.0016	0.002	0.0007	0.0091	0.0023	0.0003	0.0004	0.0066	0.0066	0.0005	0.0094	0.0008	0.0014	0.0014							
**Neoplasms**	0.0063	8.5E-07	0.0014	0.0004	2.2E-05	7.2E-05	9.5E-05		0.005				0.0024	0.0007		0.0001	0.0024	0.0044	0.0009		
**Breast Neoplasms**	0.0009	0.0047	0.0014				0.0017	0.008						0.0025		0.0093				0.0009	
**Ataxia Telangiectasia**			0.0017																		
**Adenocarcinoma**	0.0007	0.0006	0.0023	0.0042		0.0015	0.0013		0.0028	0.008		0.0053		0.004							
**Precancerous Conditio**			0.0023							0.0037											
**Adhesion**	5.2E-06	6.1E-08	0.0024	1.1E-05	3.4E-07	2.9E-10	5.4E-10	7.8E-05	1.6E-05	0.001	1.8E-05	3E-06	3.5E-08	3.5E-07						0.0091	0.0019
**Mental Disorders**		0.0003	0.0024	8.5E-06		0.0026			0.0025		0.0008									0.0049	
**Neoplasm of upecified nature of digestive system**	3.9E-05	0.0016	0.0024	0.0025	0.007	0.0026	0.0057	0.0035						0.0072	0.0074					0.0049	
**Myocardial Ischemia**	0.0008		0.0024		0.0076												0.0024		0.007		
**Skin and Connective Tissue Diseases**	0.0039		0.0024		0.0013	0.0079	0.0051														
**Li-Fraumeni syndrome**			0.0024	0.0034	0.0013					0.0055						0.0089		0.0076	0.007		
**Skin Diseases**	0.0016	0.0047	0.0029				0.0036							0.004							
**Macrophage activation syndrome**			0.0029														0.0048			0.0031	
**Gastroesophageal Reflux**	0.0043	0.0017	0.0031	0.0064	0.0074	0.0004	0.0017		0.0028	0.0087	0.0031	0.0025	0.001	0.0025							
**H Syndrome**		0.002	0.0031		0.0058	2.1E-05	2.6E-05					0.0035	0.0002	0.0007							
**Smallpox**			0.0031		0.0023					0.0017											
**Wegener Granulomatosis**			0.0031																		
**Intestinal Neoplasms**	0.0009	2.8E-05	0.0054			0.0065	0.0036		0.0003					0.0097					0.0072		

e. Tepehuanos

Missense + Promoter

	MES	TEP	ZAP	MAY	NAH	TOT	TAR
**Congenital Abnormalities**	0.0016	3.5E-08	1.4E-06	2.7E-05	0.0009	2.3E-09	1.5E-06
**Adhesion**	5.2E-06	6.1E-08	0.0024	1.1E-05	3.4E-07	2.9E-10	5.4E-10
**Cancer or viral infectio**	0.0003	6.1E-08	8.8E-05	0.0004	1.7E-05	1.2E-05	5.7E-06
**Disease Susceptibility**	1.6E-06	1.2E-07	0.0004	4.9E-06	1.4E-10	0.0002	0.001
**Neoplasms**	0.0063	8.5E-07	0.0014	0.0004	2.2E-05	7.2E-05	9.5E-05
**Genetic Predisposition to Disease**	2.2E-07	6.6E-06	9.6E-05	2.9E-05	3.4E-11	6.7E-05	4.3E-05
**Nervous System Diseases**		6.9E-06	1.1E-05	4E-08		0.0043	2E-05
**Intestinal Neoplasms**	0.0009	2.8E-05	0.0054			0.0065	0.0036
**Colonic Diseases**	0.0016	4.4E-05			0.0053	0.0036	0.0017
**Colorectal Neoplasms**	0.0032	7.5E-05	0.0099		0.0074		
**Intestinal Diseases**	0.0024	8.6E-05				0.0026	0.0014
**Gastrointestinal Diseases**	0.0008	0.0001	0.0096	0.0099		0.0099	0.0003
**Gastrointestinal Neoplasms**	0.0016	0.0002					0.0042
**Pathologic Processes**	0.0003	0.0003	0.0072	0.0076	0.0008	0.0005	0.0001
**Neoplastic Processes**	0.0004	0.0003	0.0096			1.4E-05	0.0008
**Mental Disorders**		0.0003	0.0024	8.5E-06		0.0026	
**Neoplasm Metastasis**	0.0011	0.0004				0.0074	0.0017
**Sig and Symptoms**	0.0024	0.0004	0.0099	0.0025	0.0009		
**Bipolar Disorder**	0.0032	0.0004		0.0014			
**Lung Neoplasms**		0.0004	0.0004				9.5E-05
**Neurologic Manifestatio**		0.0005	0.0072	3E-06		0.0004	0.0086
**Urogenital Neoplasms**		0.0005	9.6E-05	6.4E-05			
**Adenocarcinoma**	0.0007	0.0006	0.0023	0.0042		0.0015	0.0013
**Mood Disorders**		0.0006		0.001			
**Metabolic Diseases**		0.0007					
**Nausea**		0.0009			0.0058		
**Cardiovascular Diseases**	5.2E-06	0.001	0.00009	0.0008	1.1E-06	0.0015	0.0004
**Musculoskeletal Diseases**		0.001		0.0004	0.0023	1.1E-05	4.5E-09
**Pain**		0.0012					
**Syndrome**	0.0059	0.0015	0.0084	0.0013	0.0002	1.1E-08	9.5E-05

Missense

	MES	TEP	ZAP	MAY	NAH	TOT	TAR
**Congenital Abnormalities**		1.6E-05	0.0005	0.0013	0.0014	3.2E-07	0.0004
**Adhesion**	7.8E-05	1.6E-05	0.001	1.8E-05	3E-06	3.5E-08	3.5E-07
**Cancer or viral infectio**		0.0003			0.0059	0.002	0.0002
**Disease Susceptibility**	0.001	0.0006		7.3E-07	6.8E-08	0.0058	0.0065
**Neoplasms**		0.005				0.0024	0.0007
**Genetic Predisposition to Disease**	0.0003	0.005		1.2E-05	1.9E-08	0.0016	0.0008
**Nervous System Diseases**		0.0005	0.0055	0.0021			0.0025
**Intestinal Neoplasms**		0.0003					0.0097
**Colonic Diseases**		0.0002					
**Colorectal Neoplasms**		0.0002					
**Intestinal Diseases**		0.0005					
**Gastrointestinal Diseases**		0.001					0.0093
**Gastrointestinal Neoplasms**		0.0066					0.0083
**Pathologic Processes**	0.0027	0.005			0.0004	0.0034	0.0022
**Neoplastic Processes**	0.0031	0.0028				0.0034	0.0004
**Mental Disorders**		0.0025		0.0008			
**Neoplasm Metastasis**	0.0034						0.004
**Sig and Symptoms**	0.0055				0.0059		
**Bipolar Disorder**	0.0096	0.0001		0.0021			
**Lung Neoplasms**							0.0018
**Neurologic Manifestatio**						0.0072	
**Urogenital Neoplasms**		0.0077	0.0055				
**Adenocarcinoma**		0.0028	0.008		0.0053		0.004
**Mood Disorders**		0.0002		0.0021			
**Metabolic Diseases**		0.0002					
**Nausea**							
**Cardiovascular Diseases**	0.0035	0.0077	0.0025		0.0011		
**Musculoskeletal Diseases**						0.0034	0.0004
**Pain**		0.003					
**Syndrome**				0.0031	0.0004	5.4E-06	0.0008

Promoter

	MES	TEP	ZAP	MAY	NAH	TOT	TAR
**Congenital Abnormalities**	0.0059	0.0068	0.0055			0.0089	
**Adhesion**						0.0091	0.0019
**Cancer or viral infectio**	0.0038	0.0003	0.0012	0.0035	0.0014	0.0023	
**Disease Susceptibility**	0.0028	0.0007	0.0012		0.0065		
**Neoplasms**		0.0001	0.0024	0.0044	0.0009		
**Genetic Predisposition to Disease**	0.0013	0.0016	0.0001		0.0065		
**Nervous System Diseases**			0.0024	1E-06		0.0023	0.0043
**Intestinal Neoplasms**					0.0072		
**Colonic Diseases**					0.0083		
**Colorectal Neoplasms**					0.0022		
**Intestinal Diseases**							
**Gastrointestinal Diseases**							0.0099
**Gastrointestinal Neoplasms**		0.008					
**Pathologic Processes**			0.0024				
**Neoplastic Processes**						0.0015	
**Mental Disorders**						0.0049	
**Neoplasm Metastasis**		0.0068	0.0055				
**Sig and Symptoms**				0.0049			
**Bipolar Disorder**							
**Lung Neoplasms**		0.0059	0.0024				
**Neurologic Manifestatio**			0.0048	5E-06			
**Urogenital Neoplasms**			0.0061	0.0006			
**Adenocarcinoma**							
**Mood Disorders**							
**Metabolic Diseases**							
**Nausea**							
**Cardiovascular Diseases**	0.0028			0.0049	0.0009	0.0034	0.0043
**Musculoskeletal Diseases**				0.0022	0.0065	0.0009	1E-05
**Pain**							
**Syndrome**						0.0034	

f. Tarahumaras

Missense + Promoter

	MES	TEP	ZAP	MAY	NAH	TOT	TAR
**Adhesion**	5.2E-06	6.1E-08	0.0024	1.1E-05	3.4E-07	2.9E-10	5.4E-10
**Musculoskeletal Diseases**		0.001		0.0004	0.0023	1.1E-05	4.5E-09
**Muscular Diseases**				0.0086	0.0008	0.0006	4.9E-07
**Collagen Diseases**						0.0004	6.6E-07
**Congenital Abnormalities**	0.0016	3.5E-08	1.4E-06	2.7E-05	0.0009	2.3E-09	1.5E-06
**Cancer or viral infectio**	0.0003	6.1E-08	8.8E-05	0.0004	1.7E-05	1.2E-05	5.7E-06
**Nervous System Diseases**		6.9E-06	1.1E-05	4E-08		0.0043	2E-05
**Carcinoma**	0.0039	0.002	0.0054	0.0011	0.0013	0.0005	2.3E-05
**H Syndrome**		0.002	0.0031		0.0058	2.1E-05	2.6E-05
**Genetic Predisposition to Disease**	2.2E-07	6.6E-06	9.6E-05	2.9E-05	3.4E-11	6.7E-05	4.3E-05
**Muscular Dystrophies**				0.0015		0.0065	6.5E-05
**Heart Diseases**	0.0024	0.0073	0.0054	0.0005	2.6E-05	0.0002	9.5E-05
**Lung Neoplasms**		0.0004	0.0004				9.5E-05
**Neoplasms**	0.0063	8.5E-07	0.0014	0.0004	2.2E-05	7.2E-05	9.5E-05
**Syndrome**	0.0059	0.0015	0.0084	0.0013	0.0002	1.1E-08	9.5E-05
**Pathologic Processes**	0.0003	0.0003	0.0072	0.0076	0.0008	0.0005	0.0001
**Fetal Diseases**							0.0002
**Gastrointestinal Diseases**	0.0008	0.0001	0.0096	0.0099		0.0099	0.0003
**Neuromuscular Diseases**			0.0064	0.0034		0.0036	0.0003
**Respiratory Tract Diseases**	0.0055				0.0038		0.0003
**Cardiomyopathies**	0.0063				0.0012	0.0096	0.0004
**Cardiomyopathy NOS**	0.0063				0.0012	0.0096	0.0004
**Cardiovascular Diseases**	5.2E-06	0.001	0.00009	0.0008	1.1E-06	0.0015	0.0004
**Deglutition Disorders**	0.0016	0.002	0.0007	0.0091	0.0023	0.0003	0.0004
**Urinary Bladder Neoplasms**		0.002	0.0064				0.0004
**Vascular Diseases**	5.6E-07	0.0064	1.1E-05	5E-05	0.00002		0.0004
**Sarcoma**	0.0001						0.0007
**Neoplastic Processes**	0.0004	0.0003	0.0096			1.4E-05	0.0008
**Bone Diseases**						0.0088	0.0009
**Skin Diseases, Vascular**				0.0086			0.0009

Missense

	MES	TEP	ZAP	MAY	NAH	TOT	TAR
**Adhesion**	7.83E-05	0.000016	0.001	1.83E-05	2.98E-06	3.51E-08	3.54E-07
**Musculoskeletal Diseases**						0.0034	0.0004
**Muscular Diseases**							0.0004
**Collagen Diseases**					0.0092	0.001	3.31E-08
**Congenital Abnormalities**		0.000016	0.0005	0.0013	0.0014	3.2E-07	0.0004
**Cancer or viral infectio**		0.0003			0.0059	0.002	0.0002
**Nervous System Diseases**		0.0005	0.0055	0.0021			0.0025
**Carcinoma**					0.0066	0.0023	0.0003
**H Syndrome**					0.0035	0.0002	0.0007
**Genetic Predisposition to Disease**	0.0003	0.005		1.23E-05	1.91E-08	0.0016	0.0008
**Muscular Dystrophies**							0.0025
**Heart Diseases**				0.0031		0.0034	0.0097
**Lung Neoplasms**							0.0018
**Neoplasms**		0.005				0.0024	0.0007
**Syndrome**				0.0031	0.0004	5.42E-06	0.0008
**Pathologic Processes**	0.0027	0.005			0.0004	0.0034	0.0022
**Fetal Diseases**							0.0018
**Gastrointestinal Diseases**		0.001					0.0093
**Neuromuscular Diseases**							
**Respiratory Tract Diseases**					0.0004		0.005
**Cardiomyopathies**							0.004
**Cardiomyopathy NOS**							0.004
**Cardiovascular Diseases**	0.0035	0.0077	0.0025		0.0011		
**Deglutition Disorders**	0.0066	0.0066	0.0005	0.0094	0.0008	0.0014	0.0014
**Urinary Bladder Neoplasms**		0.0091					0.0025
**Vascular Diseases**	0.0004		0.0017		0.0014		
**Sarcoma**							0.0025
**Neoplastic Processes**	0.0031	0.0028				0.0034	0.0004
**Bone Diseases**							
**Skin Diseases, Vascular**							

Promoter

	MES	TEP	ZAP	MAY	NAH	TOT	TAR
**Adhesion**						0.0091	0.0019
**Musculoskeletal Diseases**				0.0022	0.0065	0.0009	0.000013
**Muscular Diseases**				0.002	0.002	0.0016	0.0024
**Collagen Diseases**							
**Congenital Abnormalities**	0.0059	0.0068	0.0055			0.0089	
**Cancer or viral infectio**	0.0038	0.0003	0.0012	0.0035	0.0014	0.0023	
**Nervous System Diseases**			0.0024	1.16E-06		0.0023	0.0043
**Carcinoma**			0.0055				
**H Syndrome**							
**Genetic Predisposition to Disease**	0.0013	0.0016	0.0001		0.0065		
**Muscular Dystrophies**				7.12E-06		0.0023	0.0072
**Heart Diseases**					0.0011		0.0035
**Lung Neoplasms**		0.0059	0.0024				
**Neoplasms**		0.0001	0.0024	0.0044	0.0009		
**Syndrome**						0.0034	
**Pathologic Processes**			0.0024				
**Fetal Diseases**							
**Gastrointestinal Diseases**							0.0099
**Neuromuscular Diseases**				0.0008	0.0079	0.0023	0.0035
**Respiratory Tract Diseases**							
**Cardiomyopathies**							
**Cardiomyopathy NOS**							
**Cardiovascular Diseases**	0.0028			0.0049	0.0009	0.0034	0.0043
**Deglutition Disorders**							
**Urinary Bladder Neoplasms**							
**Vascular Diseases**	0.0028		0.0024	0.0002	0.0065		
**Sarcoma**	0.0028						
**Neoplastic Processes**						0.0015	
**Bone Diseases**							
**Skin Diseases, Vascular**							

g. Mestizos

	Missense + Promoter							Missense							Promoter						
	MES	TEP	ZAP	MAY	NAH	TOT	TAR	MES	TEP	ZAP	MAY	NAH	TOT	TAR	MES	TEP	ZAP	MAY	NAH	TOT	TAR
**Genetic Predisposition to Disease**	2.2E-07	6.6E-06	9.6E-05	2.9E-05	3.4E-11	6.7E-05	4.3E-05	0.0003	0.005		1.2E-05	1.9E-08	0.0016	0.0008	0.0013	0.0016	0.0001		0.0065		
**Vascular Diseases**	5.6E-07	0.0064	1.1E-05	5E-05	0.00002		0.0004	0.0004		0.0017		0.0014			0.0028		0.0024	0.0002	0.0065		
**Disease Susceptibility**	1.6E-06	1.2E-07	0.0004	4.9E-06	1.4E-10	0.0002	0.001	0.001	0.0006		7.3E-07	6.8E-08	0.0058	0.0065	0.0028	0.0007	0.0012		0.0065		
**Hypertension**	4.5E-06				0.0012			0.0001				0.0053									
**Adhesion**	5.2E-06	6.1E-08	0.0024	1.1E-05	3.4E-07	2.9E-10	5.4E-10	7.8E-05	1.6E-05	0.001	1.8E-05	3E-06	3.5E-08	3.5E-07						0.0091	0.0019
**Cardiovascular Diseases**	5.2E-06	0.001	0.00009	0.0008	1.1E-06	0.0015	0.0004	0.0035	0.0077	0.0025		0.0011			0.0028			0.0049	0.0009	0.0034	0.0043
**Neoplasm of upecified nature of digestive system**	3.9E-05	0.0016	0.0024	0.0025	0.007	0.0026	0.0057	0.0035						0.0072	0.0074					0.0049	
**Sarcoma**	0.0001						0.0007							0.0025	0.0028						
**Coronary Artery Disease**	0.0002				0.0064			0.0055											0.0065		
**Coronary Disease**	0.0002				0.0064			0.0055											0.0065		
**Cancer or viral infectio**	0.0003	6.1E-08	8.8E-05	0.0004	1.7E-05	1.2E-05	5.7E-06		0.0003			0.0059	0.002	0.0002	0.0038	0.0003	0.0012	0.0035	0.0014	0.0023	
**Pathologic Processes**	0.0003	0.0003	0.0072	0.0076	0.0008	0.0005	0.0001	0.0027	0.005			0.0004	0.0034	0.0022			0.0024				
**Neoplastic Processes**	0.0004	0.0003	0.0096			1.4E-05	0.0008	0.0031	0.0028				0.0034	0.0004						0.0015	
**Myocardial Infarction**	0.0005				2.1E-05			0.0023				0.0002									
**Adenocarcinoma**	0.0007	0.0006	0.0023	0.0042		0.0015	0.0013		0.0028	0.008		0.0053		0.004							
**Mouth Neoplasms**	0.0007						0.0072	0.0066													
**Nervous System Malformatio**	0.0007					0.0003							0.0016		0.0059		0.0048				
**Gastrointestinal Diseases**	0.0008	0.0001	0.0096	0.0099		0.0099	0.0003		0.001					0.0093							0.0099
**Myocardial Ischemia**	0.0008		0.0024		0.0076												0.0024		0.007		
**Neoplasm Invasiveness**	0.0008		0.0099			0.0002							0.0065							0.0091	
**Rectal Neoplasms**	0.0008																				
**Breast Neoplasms**	0.0009	0.0047	0.0014				0.0017	0.008						0.0025		0.0093				0.0009	
**Infarction**	0.0009				0.0002		0.0036	0.0089				0.0025									
**Intestinal Neoplasms**	0.0009	2.8E-05	0.0054			0.0065	0.0036		0.0003					0.0097					0.0072		
**Cerebral Infarction**	0.0009			0.0005	0.0038		0.0086	0.0089				0.0059									
**Stroke**	0.0009			0.0005	0.0013		0.0086	0.0089				0.0014									
**Stroke NOS**	0.0009			0.0005	0.0013		0.0086	0.0089				0.0014									
**Hyperteion, Renal**	0.0009																				
**Kidney Diseases**	0.0009			0.0079	2.6E-06			0.0019				1.7E-05									

Supplementary Figure 4. Pathway Enrichment Analysis. Annotation matrix of disease terms enriched in different populations (MES, Mestizo; TEP, Tepehuano; ZAP, Zapoteca; MAY, Maya; NAH, Nahua; TOT, Totonaca and TAR, Tarahumara) shown as a heatmap. Dark colors indicate significantly enriched ($P<0.05$) and light colors indicate not significantly enriched disease terms ($P \geq 0.05$). The hypergeometric test (WebGestalt) was used to assess disease term enrichment, *P*-values were adjusted using Benjamini & Hochberg method and ordered according to the significance for Maya (a), Totonaca (b), Nahua (c), Zapoteca (d), Tepehuano (e), Tarahumara (f) and Mestizo (g) populations.

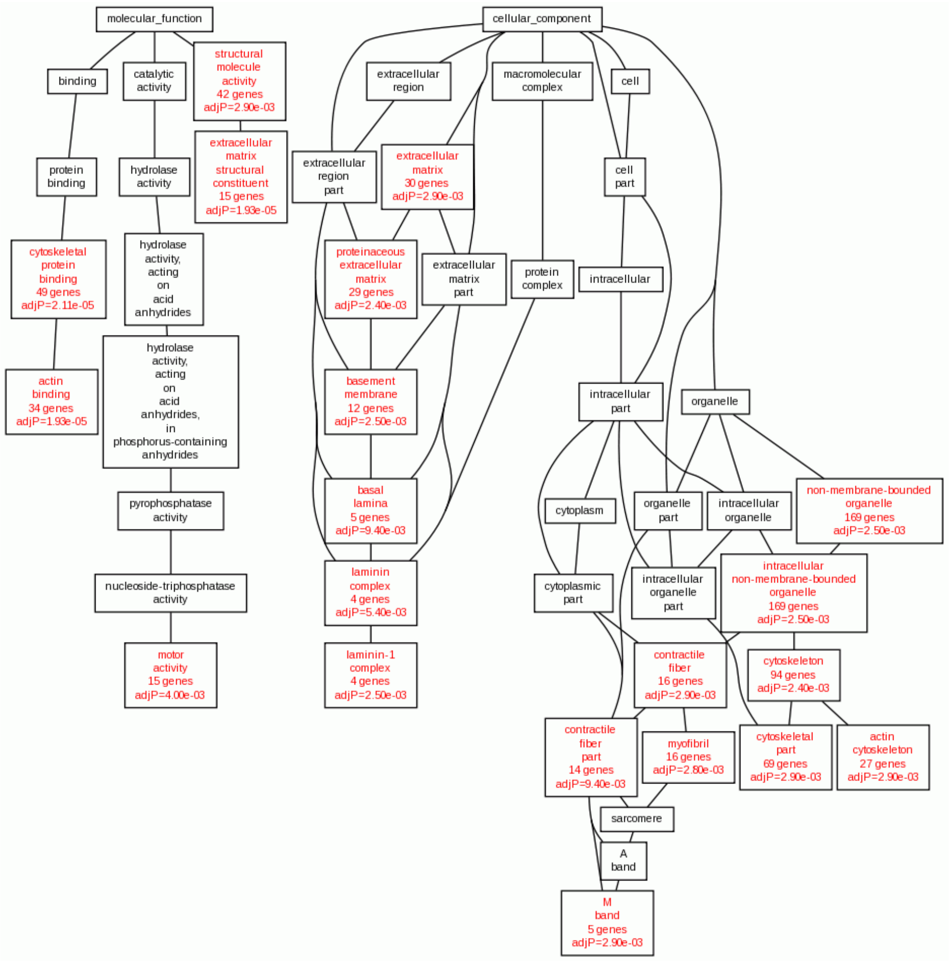

Supplementary Figure 5. Analysis of Gene Ontology (GO) Enrichment in Tarahumaras. Novel missense and novel promoter SNVs found in "molecular function" and "cellular component" pathways were tested for GO term enrichment. The resulting enriched GO terms are shown using graphic data visualization, where terms colored in red are significantly overrepresented ($P<0.05$). The hypergeometric test (WebGestalt) was used to assess GO term enrichment; *P*-values were adjusted using the Benjamini & Hochberg method.

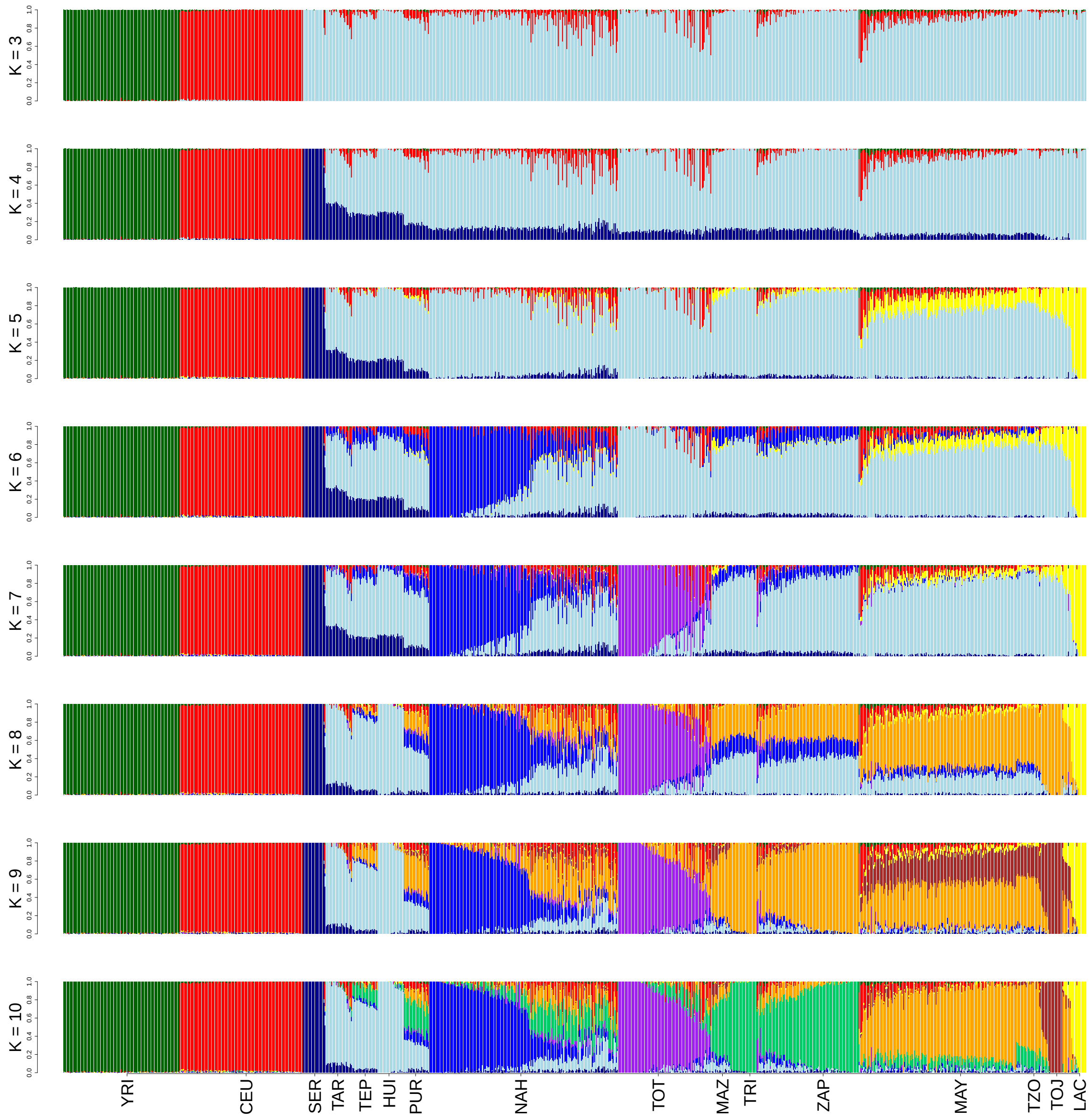

Supplementary Figure 6. Population Structure Analysis. ADMIXTURE results from k=3 to 10 based on 322,098 SNPs from 931 samples. The analysis includes 106 YRI and 112 CEU from the HapMap project; 401 Native Mexican samples from Moreno-Estrada et al. 2014[1] and 312 new Native American samples. Each vertical bar represents an individual and the y-axis represents the proportion of the genome assigned to each of the ancestral clusters.

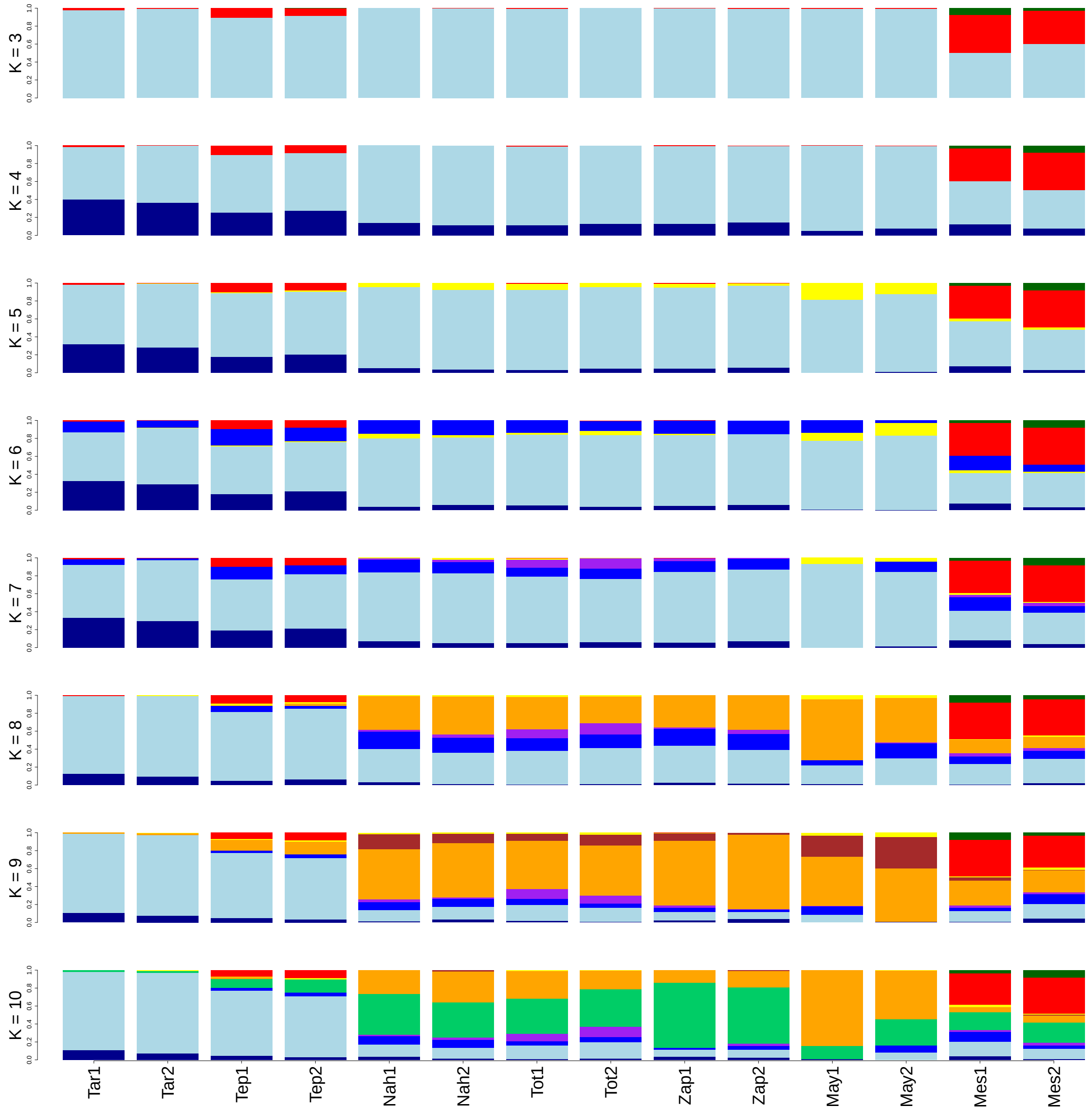

Supplementary Figure 7. Population Structure Analysis of the 15 genomes. ADMIXTURE results from k=3 to 10 based on 322,098 SNPs for the 12 Native American and 3 unrelated mestizo individuals. A total of 112 CEU, 106 YRI and 713 Native American genotypes were included as reference populations.

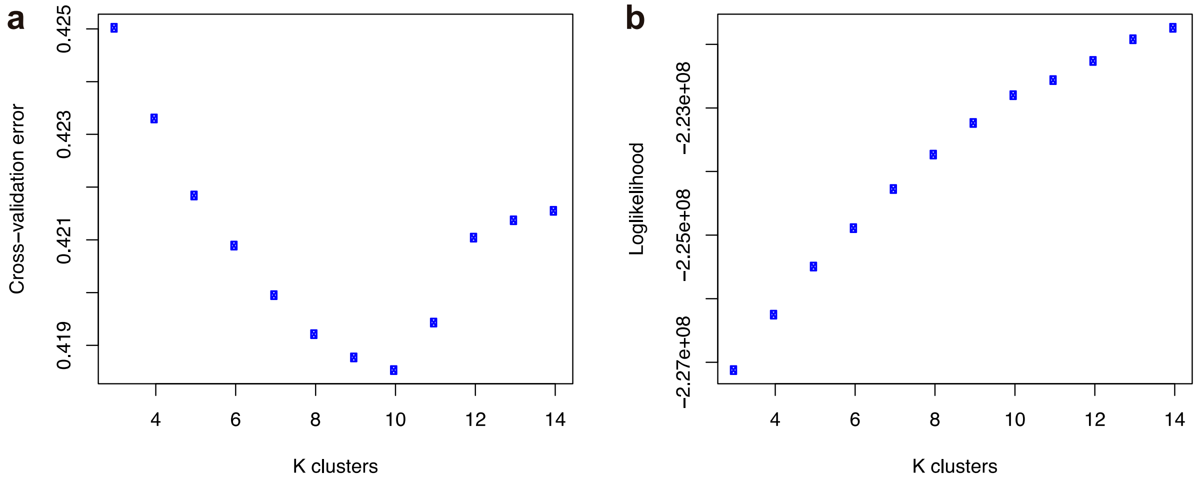

Supplementary Figure 8. Fit of different values of K using ADMIXTURE. (a) Cross-validation errors and (b) Log-likelihoods for K=3 to K=14.

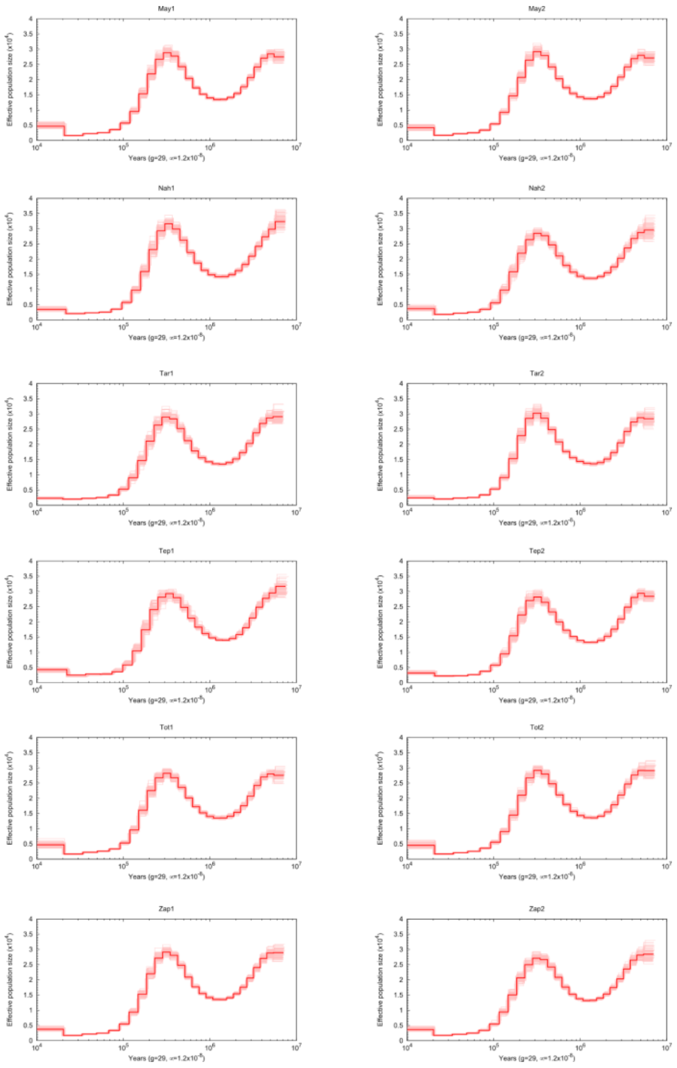

Supplementary Figure 9. Bootstrapped PSMC curves for each of the 12 NA individuals. Bootstrap results are indicated with thin lines based on replicates. One hundred bootstrap replicates were performed by randomly sampling with replacement of 5-Mb sequence segments obtained from the consensus genome sequence.

Supplementary Reference

1. Moreno-Estrada, A. *et al.* Human genetics. The Genetics of Mexico Recapitulates Native American Substructure and Affects Biomedical Traits. *Science* **344**, 1280-1285 (2014).
